# *Ishige okamurae* Suppresses Trimethyltin-Induced Neurodegeneration and Glutamate-Mediated Excitotoxicity by Regulating MAPKs/Nrf2/HO-1 Antioxidant Pathways

**DOI:** 10.3390/antiox10030440

**Published:** 2021-03-12

**Authors:** Oh Yun Kwon, Seung Ho Lee

**Affiliations:** Department of Nano-Bioengineering, Incheon National University, 119 Academy-ro, Incheon 22012, Korea; ohyun1220@naver.com

**Keywords:** *Ishige okamurae*, neurodegenerative disease, oxidative stress, glutamate excitotoxicity, trimethyltin

## Abstract

Many neurodegenerative diseases have several similar cellular dysregulations. We investigated the inhibitory role of *Ishige okamurae*, an edible brown alga, on neurodegenerative processes by estimating the effects of *Ishige okamurae* on excitotoxicity induced by glutamate in vitro and neurodegeneration induced by trimethyltin (TMT) in vivo. This study aimed to describe the molecular mechanisms responsible for the mediating anti-neurodegenerative effects of *Ishige okamurae* extract (IOE). The oral administration of IOE to TMT-injected mice impeded the TMT-mediated short- and long-term memory impairments investigated by the Morris water maze and Y-maze test. IOE attenuated TMT-mediated cellular apoptosis and the expression of brain-derived neurotrophic factor, nuclear factor erythroid 2-related factor 2 (Nrf2), and heme oxygenase-1 (HO-1) in mice brains. Glutamate-induced apoptosis and the expression of reactive oxygen species, Nrf2, and HO-1 in HT22 cells were also attenuated by IOE. In addition, TMT- and glutamate-induced phosphorylation of mitogen-activated protein kinases (MAPKs) in mouse brain tissues and HT22 cells were attenuated by the treatment of IOE. In HT22 cells, administration of MAPK inhibitors recovered the glutamate induced by the expression of Nrf2, HO-1, and cellular dysregulation to the equal extent to IOE administration. Taken together, these results suggest that IOE could attenuate neurodegenerative processes, such as TMT- and glutamate-mediated neuronal dysregulation, by regulating MAPKs/Nrf-2/HO-1 antioxidant pathways.

## 1. Introduction

Neurodegeneration is the progressive functional or structural loss of neurons that results in many incurable neurodegenerative diseases, such as Parkinson’s disease, amyotrophic lateral sclerosis, Huntington’s disease, fatal familial insomnia, and Alzheimer’s disease. These neurodegenerative diseases have been proven to have many similar phenotypes on a sub-cellular level that give us a chance to develop therapeutics to ameliorate the neurodegenerative diseases simultaneously. For this reason, several model systems, such as TMT-induced neurodegeneration and glutamate excitotoxicity, are often used for investigating the effects of therapeutic candidates on neurodegenerative diseases.

Trimethyltin (TMT) is an organotin compound used widely in the agricultural and industrial fields [1]. The systemic administration of TMT to humans induces neurodegeneration leading to clinical symptoms, such as hyperactivity and cognitive impairment, in the central nervous system (CNS) [2]. Therefore, TMT has been used as a potential model neurotoxin for investigating brain dysfunction and neurodegeneration. Several underlying mechanisms of TMT-mediated neurotoxic effects have been suggested, including intracellular calcium overload [3], glutamate excitotoxicity [4], and oxidative stress [5]. However, the detailed cellular and molecular mechanisms of TMT-induced neurodegeneration still need to be elucidated.

Glutamate plays an important role in the CNS as an excitatory neurotransmitter. However, excessive glutamate causes critical neuronal dysfunction and neurodegeneration that leads to a condition known as glutamate excitotoxicity. Chronic neurodegenerative disorders, such as amyotrophic lateral sclerosis and Parkinson’s disease, exhibit elevated glutamate levels with neuronal damage [6]. Although the precise mechanisms underlying glutamate excitotoxicity are still too complex to fully understand, many studies have suggested that oxidative stress is one of the major causes of glutamate-mediated neuronal damage [6,7,8]. Therefore, attenuation of oxidative stress by regulating the expression of molecules that control oxidative responses could be an effective way to prevent TMT- and glutamate-mediated neurodegenerative processes.

*Ishige okamurae* is a kind of edible brown algae extensively spread throughout East Asia [9]. Brown algae, including *Ishige okamurae*, are attracting attention as functional materials used in medicinal foods, since their physiological activity has been suggested to include hypoglycemic [10], anti-inflammatory [11], and antiviral effects [12]. Interestingly, it was reported that the oral administration of *Ishige okamurae* extract (IOE) for anti-Alzheimer’s disease (AD) activity suppressed the cognitive deficits and neuronal damage mediated by amyloid beta peptide (Aβ) [13]. This suggested that IOE is likely to be widely used for other neurodegenerative diseases. Therefore, in this study, we investigated whether IOE could be applied to neurodegenerative diseases other than AD by using a TMT-injected animal model and glutamate excitotoxicity in vitro. In addition, we focused on the molecular mechanisms pertaining to how IOE regulated TMT- and glutamate-induced neurodegenerative processes.

## 2. Materials and Methods

### 2.1. Preparation of IOE

IOE was prepared according to the method of Kwon Oy et al. [13]. Briefly, 70% ethyl alcohol was used for extraction of *Ishige okamurae* and the supernatant was concentrated with a vacuum evaporator (Heidolph Instruments GmbH & Co., Schwabach, Germany) and lyophilized with a freeze dryer (ilShinBioBase, Seoul, Korea). The yield was calculated as 13.7% (*w*/*w*).

### 2.2. Dosage Information

Mice were fed with IOE at a dose of 20 mg/kg bw/day over 3 weeks as described below. The human equivalent dose was calculated by using the body surface area [14], and the concentration of IOE (20 mg/kg bw/day) in TMT-injected mice could be extrapolated to human consumption as 36.45 mg/60 kg bw/day. This dosage in humans (36.45 mg/60 kg bw/day) could be fulfilled by available supplements.

### 2.3. Animals

Four-week-old male C57BL/6 mice were obtained from NARA Biotec (Seoul, South Korea) and were housed in cages that regulated the light cycle (12-h light/12-h dark), temperature (23 ± 3 °C), and humidity (55 ± 10%). Mice were allowed free access to standard food and water ad libitum (Orient Bio, Seoul, South Korea). The mice were acclimated for 1 week and then divided into 3 groups: Control (vehicle-injected, *n* = 5), TMT (TMT-injected, *n* = 5), TMT + IOE (TMT injection + oral IOE gavage at 20 mg/kg bw/day, *n* = 5). The TMT + IOE group was treated with IOE for 21 days. TMT (Sigmaldrich, Seoul, South Korea) was injected intraperitoneally into the mice (2.5 mg/kg/bw) only once after finishing the IOE administration. Control mice were inoculated with equal volumes of phosphate-buffered saline (PBS). All experimental procedures were performed according to the Incheon National University Guidelines for the Care and Use of Laboratory Animals and it was approved by the Institutional Animal Care and Use Committee of the Incheon National University (INU-ANIM-2018-11).

### 2.4. Y-Maze Test

**Figure 1:**
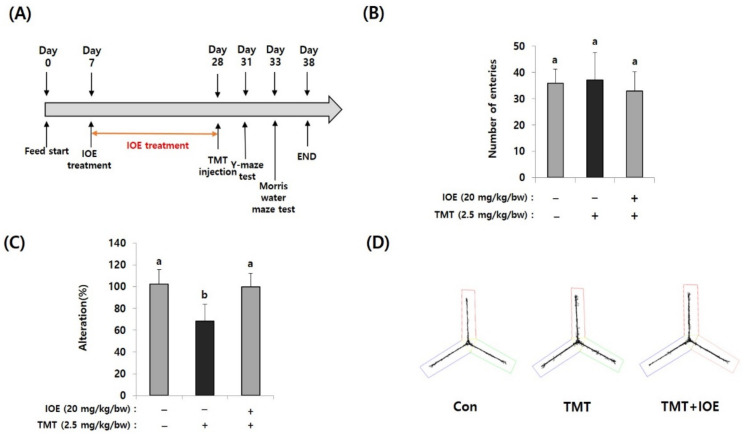
Oral administration of *Ishige okamurae* extract (IOE) attenuated the trimethyltin (TMT)-mediated spatial memory impairment in mice. After oral administration of IOE to mice (male, C57BL/6), TMT was intraperitoneally injected to mice (*n* = 5) (**A**). Memory impairment was investigated by the Y-maze test. The number of entries was not different among groups (**B**) but TMT-induced spontaneous alteration (%) was restored in IOE mice compared with that in TMT mice group (**C**). Path tracing of each group (**D**). Data shown are means *±* SD. Con: control (non-treated), a: significant differences (*p* < 0.05) when compared with b.

The Y-maze test was performed according to the method of Kwon Oy et al. [13]. Briefly, 3 days after TMT injection, the Y-maze test was started (Figure 1A). Each mouse was placed at the end of one arm and allowed to move freely through the maze for 8 min. The sequence of arm entries was manually recorded by using a SMART 3.0 video-tracking system (Harvard Apparatus, Holliston, MA, USA). Alternation was calculated by counting the number of successive entries into the arms in triplet sets. When an animal first entered A, then B, then C, this would count as one alternation (actual alternations), but an animal that entered B, then A, then B would not count as alternation. Possible alternations = total number of arm entries − 2. The alternation behavior (%) was calculated as: alternation behavior (%) = (actual alternations)/(possible alternations) × 100.

### 2.5. Morris Water Maze Test

The Morris water maze (MWM) test was carried out as described in Kwon Oy et al. [13]. The equipment consisted of a circular pool (90 cm in diameter and 60 cm high) filled with white ink (Wilton Industries, Inc., Woodridge, IL, USA) in water (22 ± 2 °C) up to 30 cm high that was divided into 4 equal quadrants. A white escape platform was placed in the center of the W zone. The mice were allowed to swim, and the latency time was monitored up to 60 s. They were permitted to stay on the platform for 5 s. In the training session (5 days after TMT injection), the mice were required to swim to escape during 3 trials per day. The probe test (10 days after TMT injection) was performed to estimate the spatial memory and long-term memory without the platform for 60 s, and the time spent in the W zone was recorded by using a SMART 3.0 video-tracking system. (Harvard Apparatus, Holliston, MA, USA).

### 2.6. Hematoxylin–Eosin Staining

Brain tissue was preserved in a formaldehyde solution (10% *w*/*v*) for tissue fixation. Tissue sections (5 μm) were subjected to hematoxylin–eosin (H&E) to observe the hippocampal regions. The number of neurons and nuclear clumping-positive cells were counted under a microscope (Nikon, Tokyo, Japan).

### 2.7. Determination of Cell Viability

HT22 cells were purchased from American Type Culture Collection (ATCC) and cultured with high-glucose Dulbecco’s modified Eagle medium (DMEM) (HyClone, Logan, UT, USA) containing 10% fetal bovine serum (FBS) (Corning, NY, USA), and 1% streptomycin/penicillin at 37 °C in a CO_2_ incubator. The HT22 cells (10^4^ cells/well) were seeded in a 96-well plate and cultured for 24 h. IOEs (0–0.1 mg/mL) were treated for 2 h and glutamate (5 mM) was then added to the culture plate. It was further incubated for 14 h at 37 °C in a CO_2_ incubator. The WST-1 Assay Kit (Dozen, Seoul, Korea) was used for measuring the cell viability and HT22 cells were photographed using microscopes (Nikon, Tokyo, Japan), and the number of viable HT22 cells was estimated.

### 2.8. Reactive Oxygen Species Measurements

The HT22 cells (10^4^ cells/well) were cultured in a black plate (96-well, SPL Life Sciences, Gyeonggi, Korea) for 24 h. HT22 cells were treated with glutamate (5 mM) and further incubated at 37 °C for 16 h with IOE or mitogen-activated protein kinase (MAPK) inhibitors (SP600125, Santa Cruz, Dallas, TX, USA; SP600125 and PD98059, Cell Signaling, Boston, MA, USA). After washing with phosphate-buffered saline (PBS), 30 μM of 2′-7′dichlorofluorescin (DCFH-DA) solution (Sigma-Aldrich Co., St. Louis, MO, USA) was added and further incubated for 1 h at 37 °C in a CO_2_ incubator. Each well was washed with PBS 2 times and then PBS (100 μL) was added to each well. The intensity of fluorescence was counted at 488 nm by using a microplate fluorometer (Tecan, Mannedorf, Switzerland).

### 2.9. Real-Time Polymerase Chain Reaction

HT22 cells (10^5^/wells) were seeded in 6-well plates and cultured for 24 h. After incubation with glutamate (5 mM) at 37 °C for 16 h with IOE or inhibitors (PD98059, SP600125, and SB203580), total RNA was isolated by using TRIzol Reagent (Invitrogen, Waltham, MA, USA). One µg of the total RNA and 10 pM of oligo dT primer were used for synthesis of complementary DNA (cDNA). SYBR Green Realtime PCR Master Mix (Toyobo Co., Tokyo, Japan) was used for the quantitative real-time polymerase chain reaction (qRT-PCR) in a RT-PCR detection system (CFX Connect, Bio-Rad Co., Hercules, CA, USA). The relative expression of each gene was estimated by using the comparative C_t_ method and normalized to the expression of glyceraldehyde 3-phosphate dehydrogenase (GAPDH). The sequences of the heme oxygenase-1 (HO-1) primers were 5′-ACATCGACAGCCCCACCA-3′ (forward) and 5′-TGAGCAGGAAGGCGGTCT-3′ (reverse), those of the Nrf-2 primers were 5′-ACTCCCAGGTTGCCCACA-3′ (forward) and 5′-TCCAGGGCAAGCGACTCA-3′ (reverse), and those of the glyceraldehyde 3-phosphate dehydrogenase primers were 5′-ACGGGAAGCTCACTGGCA-3′ (forward) and 5′-TCCAGGCGGCATGTCAGA-3′ (reverse).

### 2.10. Western Blot Analysis

Following glutamate (5 mM) administration during 16 h, after washing with PBS, the HT22 cells were lysed with a protein extraction buffer (20 mM Tris-HCl (pH 7.4), 70 μM ethylene-diamine-tetra-acetic acid (EDTA), and 150 mM NaCl, Nonidet P-40 (1%, *w*/*v*) phosphatase inhibitor cocktail (Cell Signaling, Danvers, MA, USA)) on ice for 1 h. After centrifuging (13,000× *g*) at 4 °C for 10 min, the supernatants were used for measuring each protein. At the end of the animal experiments, mouse brain tissue was separated and homogenized with a protein extraction buffer as described above by using a homogenizer (Huanyu Instrument, Zhejiang, China). Twenty μg of each protein sample was used for separating in an SDS polyacrylamide polyacrylamide gel electrophoresis (SDS-PAGE). After finishing the SDS-PAGE, the gels were separated and transferred to nitrocellulose membranes. After being incubated with 5% nonfat milk to block the nonspecific signals, membranes were incubated with each antibody (1:1500) for 12 h at 4 °C. Rabbit anti-phospho-extracellular signal-regulated kinase (ERK) (9102S), rabbit anti-ERK (9101S), rabbit anti-phospho-p38 (9212S), rabbit anti-p38 (9211S), rabbit anti-phospho- c-jun *N*-terminal kinase (JNK) (9251S), rabbit anti-JNK (9252S), rabbit anti-Bax (2772S), rabbit anti-Bcl2 (2876S), rabbit anti-cleaved caspase 3 (9664S), rabbit anti- brain-derived neurotrophic factor (BDNF) (NBP1-46750)-Novus (Centennial, CO, USA), rabbit anti-HO-1 (43966S)-Cell Signaling (Danvers, MA, USA), and mouse anti-NRF2 (M200-3) (Medical & Biological Laboratories Co., Nagoya, Japan) were used for detection of each protein. After washing with a tris-buffered saline with a Tween 20 buffer (20 mM Tris, 136 mM NaCl, (1%, *w*/*v*), Tween 20; pH 7.4), a horseradish peroxidase (HRP)-conjugated rabbit or mouse secondary antibody (1:3000) (Abcam, Cambridge, UK) was further incubated at room temperature for 2 h. The enhanced chemiluminescence (ECL) detection reagent (Bio-Rad, Hercules, CA, USA) was used for visualizing each band.

### 2.11. Statistical Analysis

All data were represented as the mean ± standard deviation (SD). The statistical analysis was performed by ANOVA using Prism 5 software (Graph-Pad Software, Inc., San Diego, CA) and a 2-tailed, unpaired Student’s *t*-test with *p* < 0.05 considered was to be statistically significant.

## 3. Results

### 3.1. TMT-Induced Cognitive Deficits Were Attenuated by Oral Administration of IOE

For investigating the effects of IOE on TMT-mediated cognitive deficits, IOE (20 mg/kg bw/day) was orally administrated for three weeks and then TMT was intraperitoneally injected into mice (Figure 1A). Since the Y-maze test is a behavioral test to measure the willingness to explore a new circumstance, it can be used to assess short-term memory in mice. As shown in Figure 1B, the number of entries was not different among groups, suggesting that TMT did not affect the basic locomotive ability of mice. However, spontaneous alteration (%) of the TMT-injected mice group (68.46 ± 15.37%) was significantly decreased (*p* < 0.05) compared with that of the control group (100.20 ± 13.50%). However, TMT did not affect the spontaneous alteration of the IOE group (99.80 ± 12.58%) (Figure 1C), suggesting that oral administration of IOE could attenuate TMT-mediated impairment of spatial working memory. In addition, we found imbalanced movement routes in TMT-injected mice compared with those of the control mice, but the IOE mice exhibited similar movement patterns to those of the control mice (Figure 1D), indicating that TMT-mediated unusual behavior could be inhibited by the oral administration of IOE.

**Figure 2:**
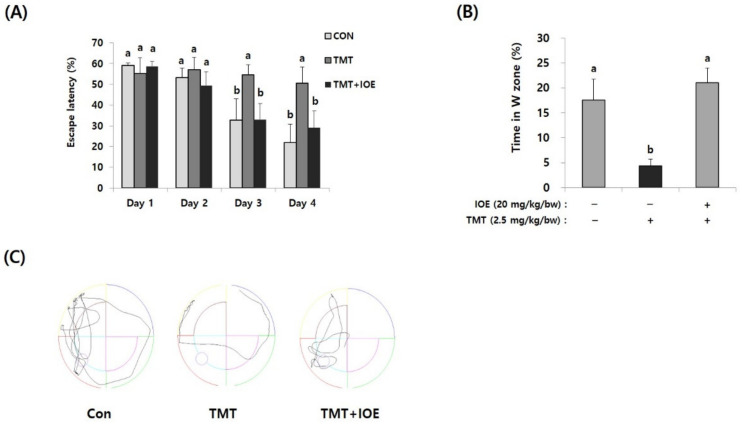
TMT-mediated impairment of long-term memory in mice was attenuated by oral administration of IOE. The effects of IOE on TMT-mediated long-term memory impairment was estimated by the Morris water maze test. Escape latency (**A**) and time in the W zone (%) at day 5 (**B**) were tabulated (*n* = 5). Movement patterns of each group during probe trials (**C**). Data shown are means ± SD. Con: control (non-treated). a: significant differences (*p* < 0.05) when compared with b.

Next, we evaluated the effects of IOE on the TMT-mediated damage to spatial and long-term learning memory by using the MWM test. As shown in Figure 2A, the time to reach the platform (escape latency) of the control mice tended to decrease during the MWM test. However, the escape latency of the TMT mice (55.71 ± 4.58%) did not decrease during the MWM test and was significantly higher (*p* < 0.05) on Day 3 of training trials compared with those of the control mice (34.93 ± 9.79%), suggesting impairment of long-term learning and spatial memory in the TMT mice. By contrast, the escape latency of the TMT + IOE mice (34.21 ± 8.05%) was significantly lower (*p* < 0.05) than that of the TMT mice (55.71 ± 4.58%) on Day 3 in the training trials, indicating that TMT-mediated impairment of long-term learning and spatial memory was attenuated by the oral administration of IOE. Furthermore, the TMT mice (4.32 ± 1.40%) stayed for shorter time periods in the W zone compared with the control mice (17.57 ± 4.21%), but the TMT + IOE mice (21.09 ± 2.87%) spent more time in the W zone compared with the TMT mice (Figure 2B,C). Taken together, these results strongly suggest that TMT-mediated cognitive deficiencies, such as memory and learning damage, could be inhibited by the oral administration of IOE.

### 3.2. TMT-Induced Neuronal Damage in Mouse Brains Was Inhibited by IOE

**Figure 3:**
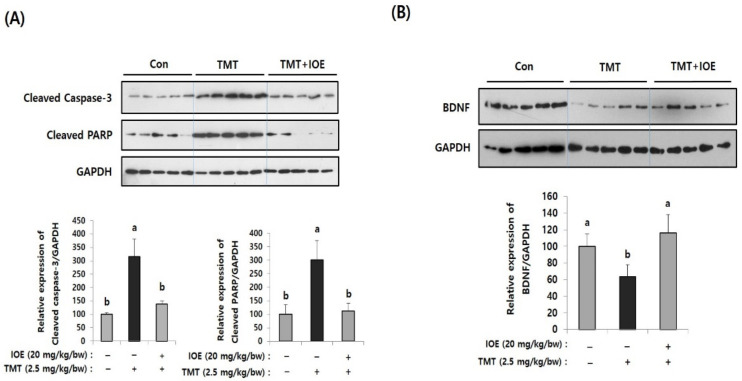
TMT-induced apoptosis in mouse brains was attenuated by oral administration of IOE. Expression of cleaved caspase-3 and cleaved poly (ADP-ribose) polymerase (PARP) (**A**) and brain-derived neurotrophic factor (BDNF) (**B**) in brain tissues of all animal groups were investigated by Western blotting (*n* = 5) (**A**). Expressions levels of each gene were estimated by measuring the intensity of each band by using the ImageJ program and were normalized to that of glyceraldehyde 3-phosphate dehydrogenase (GAPDH) and tabulated. Data shown are means ± SD. Con: control (non-treated). a: significant differences (*p* < 0.05) when compared with b.

**Figure 4:**
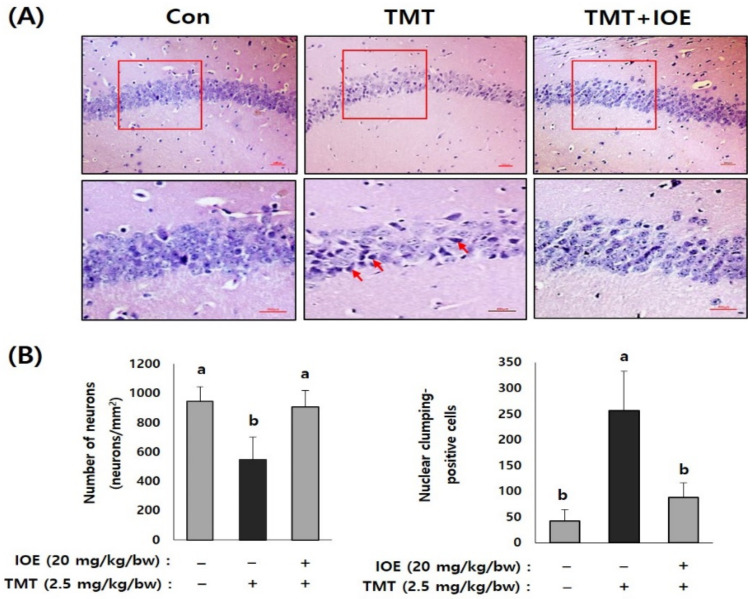
IOE attenuated TMT-induced neuronal loss in mouse brains. Representative CA1 subfield of the hippocampal region of a mouse brain stained with hematoxylin–eosin (H&E) (**A**). Enlarged area of the square in the upper panels is shown in the lower panel (**A**) and the number of viable cell and pyknotic nuclei was counted and tabulated (*n* = 5) (**B**). Arrow indicates pyknotic nuclei. Data are expressed as the means ± SD. Con: control (non-treated). a: significant differences (*p* < 0.05) when compared with b (*n* = 5). Magnification 200× (upper panel) and 400× (lower panel). Scale bar = 50 μm.

After confirming the influence of IOE on TMT-induced cognitive deficits, we evaluated the neuroprotective influences of IOE on TMT-injected mouse brains. As shown in Figure 3A, the expression of cleaved caspase-3 and cleaved poly (ADP-ribose) polymerase (PARP), markers of apoptosis, was significantly increased (*p* < 0.05) in the brains of TMT-injected mice compared with those of the control mice. Interestingly, the oral administration of IOE significantly reversed the elevated expression of cleaved capase-3 and cleaved PARP that had been detected in the TMT mice (Figure 3A). In addition, we found that the expression of the brain-derived neurotrophic factor (BDNF), which has a supportive role in the survival of existing neurons, significantly decreased (*p* < 0.05) in the brain tissue of the TMT mice compared with those of the control group. However, the expression level of BDNF in the brain tissue of the TMT + IOE mice was not significantly different (*p* > 0.05) from that of the control mice (Figure 3B). The neuroprotective effect of IOE on brain tissue was further confirmed by H&E staining. As shown in Figure 4, the number of surviving neurons in the CA1 region of the hippocampus decreased in the TMT mice compared with that of the control mice (*p* < 0.05). In addition, the number of pyknotic nuclei significantly increased (*p* < 0.05) in the CA1 region of the TMT mice compared with that of the control mice. However, these neuronal injuries significantly disappeared (*p* < 0.05) in the CA1 region of the TMT + IOE mice. Taken together, these data suggest that TMT-induced neuronal death could be attenuated by the oral administration of IOE.

### 3.3. Oral Administration of IOE-Impeded TMT-Induced Abnormal Phosphorylation of MAPKs in Mouse Brains

**Figure 5:**
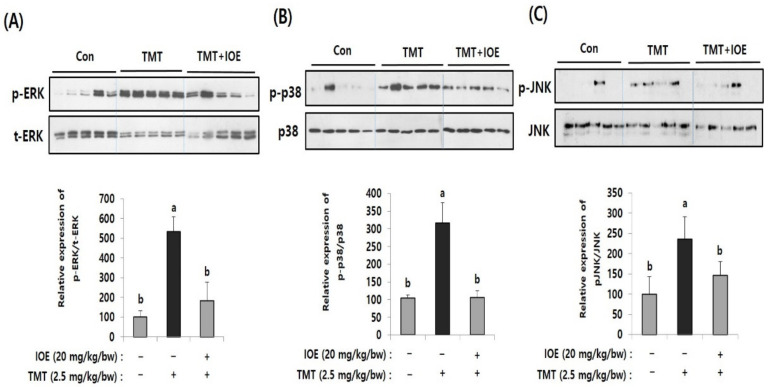
TMT-induced abnormal phosphorylation of mitogen-activated protein kinases (MAPKs) in the brains of each mouse group was restored by oral administration of IOE. Phosphorylation of extracellular signal-regulated kinase (ERK) (**A**), p-38 MAPK (**B**), and c-jun *N*-terminal kinase (JNK) (**C**) in mouse brains (*n* = 5) were measured by Western blotting. The density of each band was estimated by ImageJ program and the relative expression rates of ERK (p-ERK/t-ERK), p38 (p-p38/p38), and JNK (p-JNK/JNK) were calculated and tabulated. Data shown are means ± SD. Con: control (non-treated)). a: significant differences (*p* < 0.05) when compared with b.

MAPKs are well-known intracellular signaling molecules that control several important cellular regulations, such as apoptosis and gene expression. In addition, abnormal activation of MAPKs is reported to be closely related to neurodegenerative processes. Therefore, to elucidate the cellular mechanisms responsible for IOE-mediated neuroprotective mechanisms, we compared the effects of IOE on the activation/phosphorylation of MAPKs on the brain tissue of the mouse groups by Western blotting (Figure 5). Interestingly, we found elevated phosphorylation of extracellular signal-regulated kinase (ERK) (534.65 ± 74.80), p38 MAPK (316.96 ± 56.51), and c-jun *N*-terminal kinases (JNK) (236.48 ± 54.78) in the brain tissue of the TMT mice compared with those of the control mice (ERK: 100 ± 33.46, p38 MAPK: 100 ± 33.46, JNK: 100 ± 42.76). However, the oral administration of IOE significantly reversed (*p* < 0.05) the unusual phosphorylation of ERK, p38 MAPK, and JNK that was found in the TMT mouse group. These results suggested that TMT-mediated abnormal phosphorylation of MAPKs in mouse brain tissue could be attenuated by the oral administration of IOE.

### 3.4. TMT-Induced Abnormal Expression of Nrf2/HO-1 Could Be Attenuated by Oral Administration of IOE

**Figure 6:**
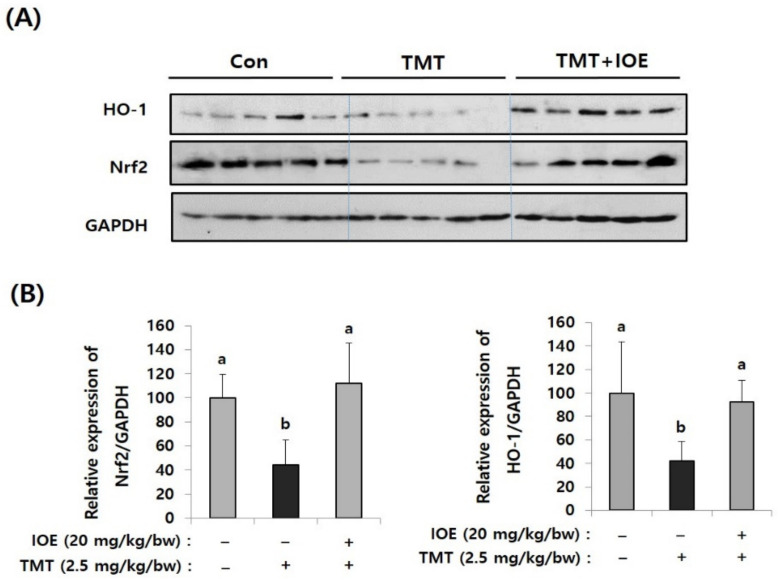
TMT-mediated suppression of nuclear factor erythroid 2-related factor 2 (Nrf2) and heme oxygenase-1 (HO-1) in mouse brain was recovered by IOE. Expression levels of Nrf2 and HO-1 in the brains of each mouse group were measured by Western blotting (*n* = 5) (**A**). The density of each band was estimated by the ImageJ program and the relative expressions of Nrf2 (Nrf2/GAPDH) and HO-1 (HO-1/GAPDH) were calculated and tabulated (**B**). Data shown are means ± SD. Con: control (non-treated). a: significant differences (*p* < 0.05) when compared with b.

Since oxidative stress has been proposed as one of the critical causes of TMT-induced neurodegenerative processes, we focused on whether the oral administration of IOE could control the antioxidant molecular systems in TMT-injected mice brains or not. Among several antioxidant mechanisms, we found that TMT-mediated abnormal expression of heme oxygenase-1 (HO-1), which has a protective role in oxidative stress, and nuclear factor erythroid 2-related factor 2 (Nrf2), a major transcriptional factor of HO-1, were restored in the TMT + IOE mice brains. As shown in Figure 6, the expression of Nrf2 and HO-1 was significantly decreased (*p* < 0.05) in the brain tissue of the TMT mouse group (Nrf2: 44.52 ± 20.28, HO-1: 41.80 ± 16.79) compared with that of the control mouse group (Nrf2: 100 ± 19.49, HO-1: 100 ± 43.92); however, it was abolished by the oral administration of IOE (Nrf2: 111.92 ± 33.50, HO-1: 92.64 ± 17.96) (*p* < 0.05). These data strongly suggested that TMT-mediated neurodegeneration may occur by blocking the intracellular signaling responsible for the expression of HO-1, which is important in cellular antioxidant processes in mouse brains. Interestingly, TMT injection increased the phosphorylation of MAPKs (Figure 5) and decreased the expression of Nrf2 and HO-1 (Figure 6). However, these TMT-induced abnormal cellular processes in mouse brains were abolished in the TMT + IOE mouse group. These data suggested that TMT-mediated oxidative stress may be inhibited by the oral administration of IOE by controlling the MAPKs/Nrf2/HO-1 pathways in mouse brains.

### 3.5. Glutamate-Mediated Excitotoxicity Was Inhibited by IOE Treatment in HT22 Cells

**Figure 7:**
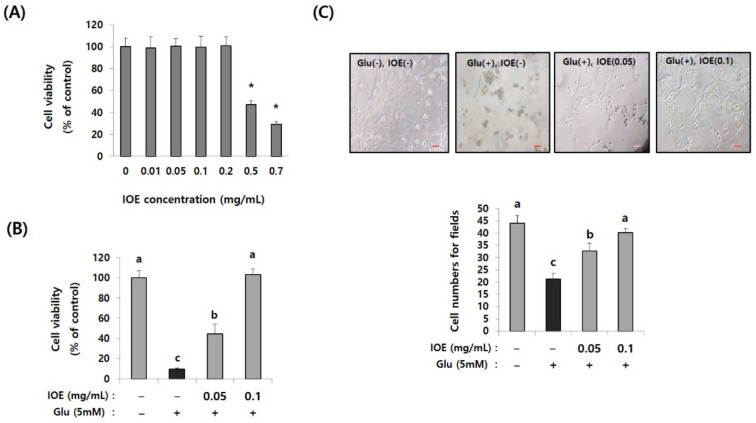
Glutamate-induced cytotoxicity was attenuated by IOE treatment. The cytotoxicity of IOE on HT22 cells was measured by a cell viability assay kit (**A**). The glutamate-mediated cytotoxicity was inhibited by the treatment with non-toxicological levels of IOE. Cell viability was measured by a WST-1 Assay Kit (**B**) and cell counting (**C**). Data value shown are means ± SD. Non-treated cells (IOE- and Glu-) were used for the control. Experiments were repeated three times with similar results. a: significant differences (*p* < 0.05) when compared with b or c; b: significant differences (*p* < 0.05) when compared with a or c, *: significant difference compared with the control (*p* < 0.01). Glu: Glutamate.

Since glutamate excitotoxicity is considered a cause of several neurodegenerative diseases responsible for initiating neuronal damage by inducing oxidative stress in neuronal cells, we further verified the IOE-derived anti-neurodegenerative effects on the cell model of glutamate excitotoxicity in our animal experiments. First, we determined the non-toxicological levels of IOE on HT22, mouse hippocampal neuronal cells by using a cell viability assay kit. The treatment of HT22 cells with IOE up to a concentration of 0.2 mg/mL could not change any HT22 cell viability (*p* < 0.05) (Figure 7A). Based on this result, glutamate-mediated cytotoxicity was dose-dependently attenuated by the IOE treatment (control: 100 ± 7.62, glutamate: 9.64 ± 1.24, glutamate + IOE 0.05: 44.53 ± 9.36, glutamate + IOE 0.1: 103.19 ± 4.27) (*p* < 0.05) (Figure 7B). The effects of IOE on glutamate-mediated cytotoxicity were further confirmed by counting the number of HT22 cells. As shown in Figure 7C, a number of viable HT22 cells were restored by using IOE administration (control: 43.93 ± 2.55, glutamate: 21.68 ± 2.27, glutamate + IOE 0.05: 32 ± 3.93, glutamate + IOE 0.1: 39.62 ± 1.78) (*p* < 0.05). These data suggest that glutamate-induced excitotoxicity in HT22 neuronal cells could be attenuated by IOE.

### 3.6. Administration of IOE Inhibited Glutamate-Mediated Reactive Oxygen Species Overproduction

**Figure 8:**
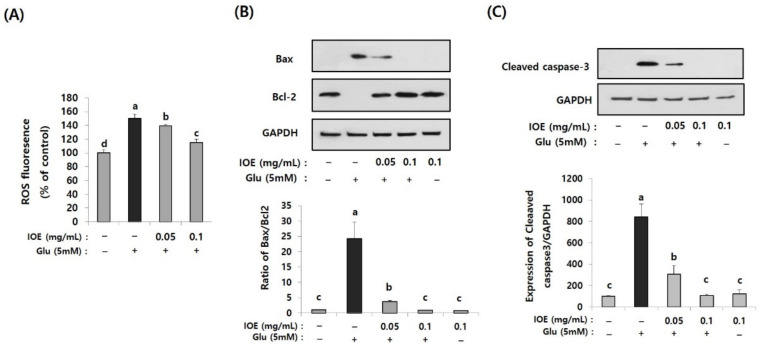
Glutamate-induced intracellular reactive oxygen species (ROS) and apoptosis were inhibited by IOE. Glutamate-mediated ROS production was dose-dependently inhibited by IOE administration in HT22 cells (**A**). Expression of apoptosis-related genes (Bax, Bcl-1, and cleaved caspase-3) were estimated by Western blotting (**B**,**C**) (*n* = 3). The density of each band was estimated by the ImageJ program, and the ratio of Bax/Bcl-2 and the relative expression of cleaved caspase-3 (cleaved caspase-3/β-actin) was calculated and tabulated. Data shown are means ± SD. Glu: Glutamate. Non-treated cells (IOE- and Glu-) were used for the control. Experiments were conducted in triplicate and repeated three times with similar results. a: significant differences (*p* < 0.05) when compared with b or c; b: significant differences (*p* < 0.05) when compared with a, c and d.

Excessive glutamate is known to cause cell stress by increasing intracellular reactive oxygen species (ROS), which causes cellular oxidative stress. The effects of IOE on glutamate-induced intracellular ROS levels were estimated in HT22 cells. As shown in Figure 8A, levels of intracellular ROS on glutamate-treated HT22 cells increased (150.24 ± 5.70) compared with those of the control group (100 ± 5.64). However, it was dose-dependently attenuated by IOE treatment (IOE 0.05: 139.14 ± 2.11, IOE 0.1: 114.76 ± 5.20) (*p* < 0.05). These results indicated that glutamate-mediated cellular stress, such as ROS overproduction, could be effectively hindered by IOE administration.

### 3.7. IOE Attenuated the Glutamate-Induced Activation of Apoptosis in HT22 Cells

Since the glutamate-mediated cytotoxicity and intracellular ROS levels were attenuated by IOE (Figure 7 and Figure 8A), we then investigated whether IOE could inhibit the glutamate-induced apoptosis of HT22 cells. The expression of Bax, a pro-apoptosis molecule, significantly increased after treating HT22 cells with glutamate (1 ± 0.0256) and it was attenuated by IOE treatment (0.901 ± 0.045) (Figure 8B). However, the expression of Bcl-2, an anti-apoptosis molecule, was attenuated by glutamate treatment (1 ± 0.0354), but it was recovered after IOE treatment (0.834 ± 0.048) (Figure 8B) (*p* < 0.05). In addition, treatment with IOE significantly attenuated (*p* < 0.05) the glutamate-induced expression of cleaved caspase-3, an intracellular apoptosis signal in HT22 cells (Figure 8C). These results suggest that IOE possesses inhibitory activity against glutamate-induced HT22 cell death, mainly by controlling the expression of apoptosis-related genes.

### 3.8. IOE Attenuated the Glutamate-Induced Activation of MAPKs in HT22 Cells

**Figure 9:**
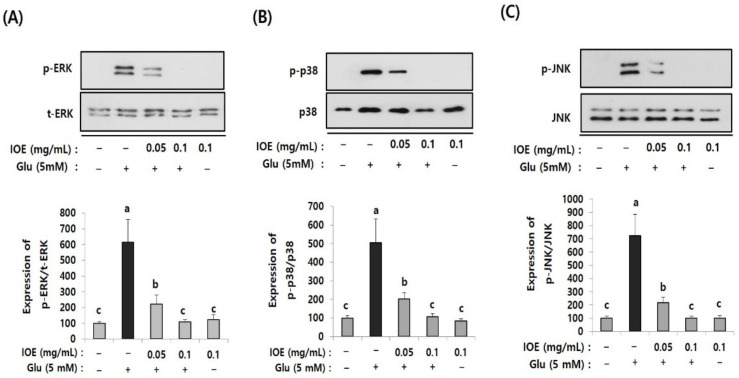
The glutamate-induced dysregulation of MAPKs was reversed by IOE. HT22 cells were pretreated with IOE for 2 h with and without glutamate (5 mM), and the phosphorylation of ERK (**A**), p38 MAPK (**B**), and JNK (**C**) was measured by Western blotting (*n* = 3). The density of each band was estimated by ImageJ program and the value of each band was calculated and tabulated. Each data value is expressed as the mean ± SD. Glu: Glutamate. Non-treated cells (IOE- and Glu-) were used for the control. Experiments were conducted in triplicate and repeated three times with similar results. a: significant differences (*p* < 0.05) when compared with b or c; b: significant differences (*p* < 0.05) when compared with a or c.

To further elucidate the regulatory role of IOE in glutamate excitotoxicity in HT22 cells, the effects of IOE on glutamate-induced intracellular activation of MAPKs were investigated. As shown in Figure 9, the treatment of HT22 cells with glutamate increased the activation/phosphorylation of ERK, p38 MAPK, and JNK proteins, just like what appeared in the brain tissue of the mice treated with TMT (Figure 5), compared with that of the control group. Interestingly, these abnormal activation/phosphorylation of MAPKs in glutamate-treated HT22 cells was dose-dependently attenuated by IOE treatments (*p* < 0.05) (Figure 9). These results suggest that glutamate-induced dysregulation of MAPKs in HT22 cells could be recovered by the treatment with IOE.

### 3.9. Glutamate-Induced Nrf2 and HO-1 Suppression Were Restored by IOE Treatment and MAPK Inhibitors

After confirming the regulatory effects of IOE on glutamate-induced abnormal activation of MAPKs, we then investigated the MAPKs’ downstream pathways associated with oxidative stress. Since oxidative stress is known as one of the important causes of glutamate excitotoxicity, and TMT-mediated Nrf2 and HO-1 expression were recovered by IOE in mouse brain tissue (Figure 6), we investigated the effects of IOE on glutamate-induced expression of Nrf2 and HO-1 in HT22 cells. In addition, we further confirmed the roles of MAPKs on glutamate-induced expression of Nrf2 and HO-1 by MAPK-specific inhibitors (SB203580, p38 MAPK inhibitor; PD98059, MEK inhibitor; SP600125, JNK inhibitor). As shown in Figure 9, glutamate-induced suppression of Nrf2 and HO-1 expression was recovered by IOE administration (*p* < 0.05). Furthermore, we found that glutamate-mediated expression of Nrf2 and HO-1 was restored by SB203580, PD98059, and SP600125 administration (*p* < 0.05). These results indicated that the glutamate-mediated suppression of HO-1 expression could be attenuated by IOE and MAPK (ERK/p38 MAPK/JNK) pathways, which are critically involved in glutamate-mediated neurodegeneration by controlling the expression of Nrf2 and HO-1.

### 3.10. Glutamate-Induced ROS Overproduction and the Attenuation of Cell Viability Were Restored by ERK, p38 MAPK, and JNK Inhibitors as Well as IOE

**Figure 10:**
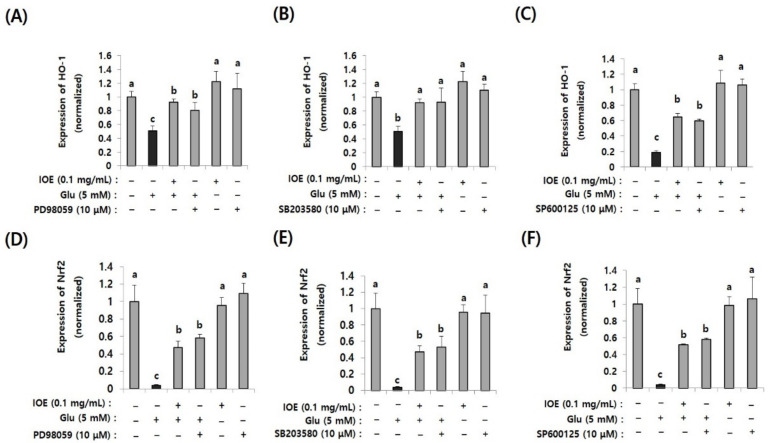
The glutamate-induced suppression of Nrf2 and HO-1 was restored by IOE (pretreatment for 2 h) as well as MAPK inhibitors (PD98059, SB203580, and SP600125; pretreatment for 1 h). The glutamate-induced expression of Nrf2 (**D**–**F**) and HO-1 (**A**–**C**) in HT22 cells was estimated by qRT-PCR (*n* = 3). The effects of PD98059 (**A**,**D**), SB203580 (**B**,**E**), and SP600125 (**C**,**F**) on the expression of Nrf2 and HO-1 were estimated by qRT-PCR (*n* = 3). Non-treated cells (IOE, Glu, and inhibitors-) were used for the control. Experiments were conducted in triplicate and repeated three times with similar results. Data shown are means ± SD. a: significant differences (*p* < 0.05) when compared with b or c; b: significant differences (*p* < 0.05) when compared with a or c.

**Figure 11:**
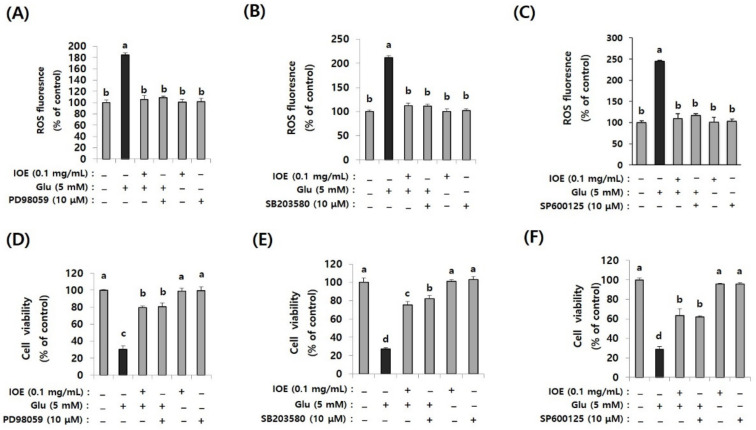
Glutamate-induced ROS overproduction and cytotoxicity in HT22 cells were restored by IOE as well as MAPK inhibitors. After pretreatment of HT22 cells with IOE (2 h) and MAPK inhibitors (1 h) (PD98059; MEK inhibitor, SB203580; p38 MAPK inhibitor, SP600125; JNK inhibitor) ROS overproduction was estimated by using a microplate fluorometer (**A**–**C**), and cell viability (**D**–**F**) was estimated by using a WST-1 Assay Kit. Non-treated cells (IOE, Glu, and inhibitors) were used for the control. Data shown are means ± SD. Glu: Glutamate. Experiments were conducted in triplicate and repeated three times with similar results. a: significant differences (*p* < 0.05) when compared with b or c, or d; b: significant differences (*p* < 0.05) when compared with a or c, or d; c: significant differences (*p* < 0.05) when compared with a or b, or d.

**Figure 12:**
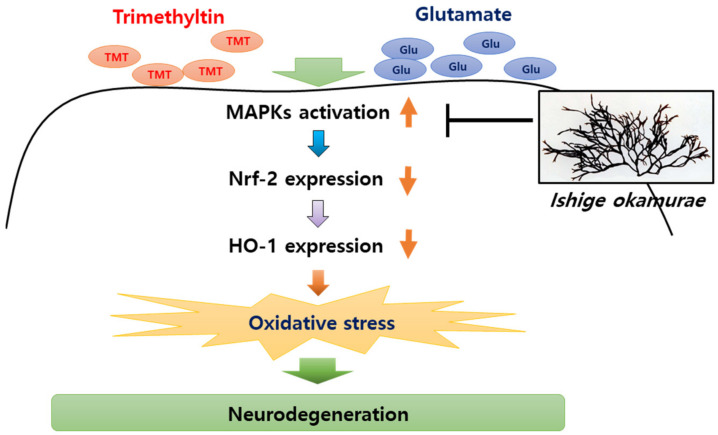
IOE-mediated anti-neurodegenerative effects. Oxidative stress in brain tissue has been considered as a major cause of neurodegenerative diseases. TMT and glutamate can induce the phosphorylation of MAPKs in the brain, and elevated phosphorylation of MAPKs attenuate the expression of Nrf2 and Heme oxygenase-1 (HO-1), resulting in oxidative stress in the brain. Our study suggested that oral administration of IOE ameliorates oxidative stress-mediated neurodegeneration through regulating the MAPK/Nrf2/HO-1 antioxidant pathways.

Because the glutamate-induced suppression of HO-1 was restored by IOE, as well as MAPK inhibitors (Figure 10), we further validated the anti-neurodegenerative effects of IOE and MAPK inhibitors on glutamate-induced ROS production and HT22 cell viability. As shown in Figure 11, glutamate-induced ROS overproduction was significantly inhibited (*p* < 0.05) by PD98059, SB203580, and SP600125 treatment (Figure 11A–C). In addition, we also found that the glutamate-mediated HT22 cell viability was significantly restored (*p* < 0.05) by the treatment with these inhibitors (Figure 11D–F). Interestingly, a 0.1 mg/mL dose of IOE had powerful inhibitory activities (as much as 10 µM of each MAPKs signal inhibitor). Taken together, these data strongly suggest that IOE was able to protect against glutamate excitotoxicity in HT22 cells by attenuating the glutamate-mediated dysregulation of MAPK/Nrf2/HO-1 pathways (Figure 12).

## 4. Discussion

Although the genetic causes of neurodegenerative diseases are various and complex, they can be characterized by progressive neuronal death. Neuronal loss from specific regions of the brain induce impairment of memory and the cognitive deficits leading to dementia. Most people suffering with neurodegenerative diseases show a loss of short- and long-term memory with neuronal damage to the hippocampal regions in the brain, which are important for learning and processing spatial memories. Therefore, the attenuation of neuronal death is able to be a crucial strategy in neurodegenerative disease prevention.

TMT is a well-known neurotoxin often used for constructing model systems of neurodegenerative diseases, since the injection of TMT can specifically induce neuronal death in the hippocampal region with cognitive deficits [2]. In this study, we showed that TMT-induced neurodegenerative processes, such as neuronal loss and memory impairment in the hippocampal region, were attenuated by the oral administration of IOE. In addition, TMT-induced neuronal apoptosis was significantly inhibited by IOE administration, suggesting that IOE has a strong potential to be developed as an agent for inhibiting neurodegenerative diseases.

BDNF is a member of the neurotrophin family of growth factors that plays an important role in neural development and cognitive function by controlling the synaptic plasticity of the brain. Since synaptic dysfunction is evidence of neurodegenerative diseases, such as Parkinson’s disease [15], Alzheimer’s disease [16], and Huntington’s disease [17], promoting the expression of BDNF to repair the synaptic loss could be a strategy for attenuating neurodegenerative disorders. In this study, we showed that TMT-mediated expression of BDNF was recovered by IOE administration in the brain, indicating that the impairment of synaptic plasticity in TMT-injected mouse brains may be attenuated by IOE administration.

Among the possible mechanisms of TMT-mediated neurotoxicity, impairment of the neurotransmitter system has been noticed as a crucial cause of TMT-induced neurodegeneration [4,18]. Neurotransmitters, such as glutamate and *N*-methyl-D-aspartic acid (NMDA), are chemical messengers that play an important role in transmitting a message from a nerve cell to a target cell. However, if the concentration of neurotransmitters exceeds pathological levels, it can induce neuronal death, known as excitotoxicity. Glutamate is a well-known neurotransmitter of neuronal signals via glutamate receptors in the brain. However, excessive levels of extracellular glutamate could induce overproduction of ROS, resulting in oxidative damage in neuronal cells. Furthermore, glutamate excitotoxicity has been proposed as a cause in TMT-mediated neurodegeneration [4,18,19]. Therefore, although the exact mechanisms of how TMT affects the expression of glutamate in the brain are still unclear, glutamate-induced oxidative stress could be a cause of TMT-mediated neuronal cell death. In this study, glutamate-induced HT22 cell death as well as ROS overproduction were attenuated by IOE treatment, indicating that IOE could effectively inhibit glutamate excitotoxicity.

One of the common pathophysiological elements in neurodegenerative diseases is oxidative stress, and excessive oxidative stress is known to be a major cause of neuronal cell death. To develop effective agents to prevent the progression of neurodegenerative diseases, many studies have focused on developing new agents that induce the expression of antioxidative molecules, including glutathione peroxidase, superoxide dismutase, and HO-1 [20,21]. Recently, HO-1 in neurodegeneration has received much more attention because it can catalyze heme to bile pigments (biliverdin and bilirubin), which have strong antioxidant properties [22,23]. Thabit S et al. reported that the administration of an extract from *Styphnolobium japonicum* (SJ) fruits to TMT-injected mouse attenuated oxidative stress by recovering the expression of HO-1 in mice brains [24]. In addition, it was reported that cerebellar granule neurons (CGSs) overexpressing HO-1 showed resistance against glutamate-induced oxidative stress [25], indicating that HO-1 expression can be a critical defense mechanism against oxidative stress-mediated neurodegeneration. Interestingly, we found that IOE administration to TMT-injected mice recovered the expression of HO-1 in mouse brains. Furthermore, IOE could restore the glutamate-mediated expression of HO-1 in HT22 neuronal cells, suggesting that IOE-mediated anti-neurodegenerative effects could be a result of attenuating the oxidative stress by regulating the expression of HO-1.

Since the expression of HO-1 was increased through the treatment of IOE in both the TMT-injected animal model and glutamate excitotoxicity cell model in our study, we investigated the intracellular signaling pathway that controlled the IOE-mediated HO-1 expression in the brain. MAPK is a central signaling pathway that controls cellular proliferation, differentiation, and apoptosis. In addition, increased evidence has been found that neurodegenerative processes are closely related to the MAPK pathways [26,27]. Furthermore, it was revealed in our previous study [13] that MAPKs play a critical role in IOE-mediated anti-AD effects, so we considered that MAPK pathways could be involved in IOE-mediated anti-neurodegenerative processes that control the expression of HO-1. In this study, we found that the expression of Nrf2, a major transcription factor that regulates the expression of HO-1, was recovered by IOE administration in the brains of TMT mice, as well as glutamate-treated HT22 cells. Furthermore, TMT- and glutamate-induced abnormal phosphorylation of MAPKs was attenuated by the oral administration of IOE in mice brains and HT22 cells, respectively. Interestingly, the administration of MAPK-specific inhibitors to glutamate-treated HT22 cells restored the glutamate-induced expression of Nrf2 and HO-1, as well as cellular dysregulation in HT22 neuronal cells, suggesting that MAPKs could be key regulators located upstream of the Nrf2-mediated transcription of HO-1 genes.

## 5. Conclusions

In this study, we evaluated the efficacy of IOE on neurodegenerative diseases by using TMT-injected animal and glutamate excitotoxicity cell systems. IOE effectively attenuated TMT- and glutamate-induced neurodegenerative events, such as cognitive deficits and neuronal death. In addition, we found a molecular mechanism of IOE mediated anti-neurodegenerative events. Although the preventive effects of IOE on the neurodegenerative events were investigated in this study, considering the fact that most drug therapies begin after disease diagnosis, the treatment effects of IOE on the neurodegenerative progress should be verified in further study. Taken together, our results strongly suggest that IOE could be used for developing anti-neurodegenerative disease agents that can control oxidative stress-mediated neurodegeneration.

## Data Availability

Data is contained within the article.
